# Sympathetic Paralysis

**Published:** 1870-07

**Authors:** H. R. Noel


### ARTICLE X.

### *Sympathetic Paralysis.*

By Prof. H. R. Noel. M. D.

Mr William F. Turner, cattle dealer, age 39, weight 194 pounds, health excellent, and of superb muscular development, March 7th, 1870, while endeavoring to open an oyster, glanced the knife, and made a deep punctured wound of the thenar region of the left hand, about the middle of the metacarpal bone of the thumb, and reaching to the bone. There was instantly a sharp arterial hemorrhage, which soon ceased, and decided pain, of an acute character, which continued. This pain crept slowly up the left forearm, then up the arm to the shoulder joint, and finally to the neck, reaching the neck in about five minutes. In an instant after the pain reached the neck, the *right hand* became acutely painful, and suddenly complete unconsciousness supervened, some slight spasm of the facial muscles, eyes open and staring, mouth relaxed and open; then a knotting o muscles and rigid tonic spasm ensued.

Involuntary discharge of urine took place. The patient regained consciousness with a feeling of intense pain in the *right hand* and none in the other *i. e.* the left hand, which was wounded; but in a few minutes the pain returned to the left also, and was equally violent in the two, right and left. Unconsciousness lasted possibly ten minutes. After the return of consciousness, as shown plainly by the eyes and by the efforts of the patient to speak, there still remained, for a few minutes, a complete loss of control over the muscles of the head and neck, and the face had a flat, relaxed, paralytic expression. There was marked pallor, nausea, trembling and great temporary prostration. The right and left hands were equally painful now, the pain involving the thumb, first, second and half of the third finger of each hand; there was unsteadiness, a sort of paralysis agitanos and numbness in each.

The numbness and pain remained, but decreasing gradually until the third day. Upon the third day the patient said his left hand was *sore*, stiff, numb or dead; his right hand ached and trembled, but was not numb. Fourth day, but little pain or numbness, and that confined to the left hand; some soreness around the wound; muscular coordination completely restored, and patient perfectly well the eigth day.

The hemorrhage was from the princeps pollicis artery, or from a branch. The pain was from a crushing of some branches of the median nerve; but the strange element was the reflected trouble in the right hand, and I am of the opinion that the true explanation of this case would also explain the following cases, viz:

Earache in children during dentition; pain in the heel from calculus in the kidney; pain in the knee-joint from disease in the hip; paralysis of the arm from carious teeth; paralysis in the lower limbs from uterine trouble.

*Query.* Are these, cases of reflex sympathetic paralysis, and of reflex sympathetic pain? If so, are there any laws or rules, physiological or pathological, by which we can distinguish them from cases of true paralysis?—*Baltimore Medical Journal.*

